# The 1990 International Symposium on Laboratory Automation and Robotics

**DOI:** 10.1155/S146392469000030X

**Published:** 1990

**Authors:** Glenn L. Taylor, Thomas R. Smith, Gregory J. Kamla

**Affiliations:** Shell Development Company Houston Texas 77251 USA


Journal of Automatic Chemistry, Vol. 12, No. 6 (November–December 1990), pp. 227–250

### The 1990 International Symposium on Laboratory Automation and Robotics

*The eighth meeting on this subject was held in Boston from 16 September to 19 September 1990, and brought together around 300 delegates mainly from the USA but with a few from other countries. Hosted by Zymark, the conference covered various facets of automation whilst focusing on laboratory robotics. The symposium included a series of plenary lectures, invited lectures and poster sessions. The highlights were, for this delegate, the first plenary by Glenn Taylor of Shell (on 'Robotics fulfill a strategic need') and a visit to the Zymark facility where several distinct robotic systems were in evidence. Whilst promoted as robotic systems these exhibits included quite a considerable degree of laboratory automation, especially solution handling.*

*Glen Taylor's plenary session set the meeting off on a high note. He outlined his company's commitment to automation and robotics and described the changes that encouraged this move, and the problems associated with the acceptance by staff etc., prior to introducing it into all applications.*

*We are in a new culture where the focus is on the customer's needs for analysis rather than just for numbers. New demands by regulatory requirements, environmental requirements, pressures from competition and, in the oil business, by a change in crude starting materials, are specific factors which have changed customers' needs. Accurate, cost-effective analyses are now of paramount importance and these require good quality control, automation and robotics. A vital role for automation is to improve the credibility of analytical results and Dr Taylor suggested that up to 30% of all analytical demands were for blind duplicates – sent to test the analyst rather than because they were needed. Better results would avoid some of this additional work cost.*

*The second plenary was presented by M. B. Fawzi, and, although missing some of the structure and enthusiasm of the first paper, it also had a number of valid points to make, especially the move from laboratory automation to direct on-line requirements in drugs and to speed up their introduction into use.*

*Following these papers some awards were made for contributions to the field of robotics.*

*The recipients were:*

Dante Rutstrom (Eastman Chemicals Company).
Gordon Plummer (Imperial Chemical Industries).
Ronald Mamajek (R. W. Johnson Pharmaceuticals).
Dennis France (Sandoz Research Institute).
Louis Lofgreen (Hercules Aerospace).
Dr Thomas Smith (Shell Development Company).
Dr Gregory Kamla (Shell Development Company).
Charles Luker (Colgate Palmolive).
Marla Elliott (Colgate Palmolive).

*A full programme of lectures and posters followed – the abstracts are included in this issue of 'Journal of Automatic Chemistry' by kind permission of the Zymark Corporation – and will be available in early 1991 as 'Advances in Laboratory Automation and Robotics' Vol. VII.*

*The meeting was extremely successful and lively. It is one that is well worth fitting into your diaries for 1991 – the ninth symposium will be held from 20 to 23 October 1991 and information is available from Sharon Correia, Zymark Corporation, Zymark Center, Hopkinton, Massachusetts 01748, USA. Tel.: 508 435 9500; Fax: 508 435 3439.*

### *Abstracts from the 1990 International Symposium on Laboratory Automation and Robotics*

### KEYNOTE

### Robotics fulfill a strategic need

*Glenn L. Taylor, Thomas R. Smith and Gregory J. Kamla (Shell Development Company, Houston, Texas 77251, USA)*

In the analytical laboratory, robotics have advanced from being a novelty to being an indispensable tool with a track record of proven success and dependability. Pressures for lower costs, faster turnaround and better quality control have created an environment in which robotics provide a unique and strategic solution. The question is no longer can a robot work, but, rather, when should a robot be used?

Productivity, precision, safety and quality-of-life improvements are commonly observed and have been well documented; however, robots are not a panacea for all situations. The job of management is to recognize applications which can benefit most from their capabilities and to understand the reasons why implementation is not always easy even when the application is appropriate. The job of scientists and technicians is to ensure that workable and practical systems are selected, developed and implemented. When both jobs are done well, the results can be dramatic.

0142–0453/90 $3.00 © 1990 Taylor & Francis Ltd.

### KEYNOTE

### Emerging trends in laboratory automation in pharmaceutical product development

*M. B. Fawzi (Warner-Lambert Company, Parke-Davis Pharmaceutical Research Division, Morris Plains, New Jersey 07950, USA)*

In the past few years, pharmaceutical development scientists have been challenged with increasingly complex products, stricter government regulations and much tougher competition. This has significantly increased the need for better and faster analytical testing capabilities. Consequently, we are witnessing the early stages of a revolution in the practice of analytical chemistry in pharmaceutical R&D laboratories. The basic elements of this revolution centre around the development of laboratory automation and the changing role of the analytical chemist in the pharmaceutical R&D process.

This presentation addressed current trends in 'Robotics', laboratory automation, LIMS and future directions for 'the intelligent laboratory'. In addition, the potential application of new areas such as 'expert systems' and 'applied chemometrics' in pharmaceutical R&D were highlighted.

---

### The use of automated gravimetric techniques for enhanced analytical precision

*Brian G. Lightbody and Sally D. Dowling (Zymark Corporation, Hopkinton, Massachusetts 01748, USA)*

The use of automated equipment for routine sample preparation is growing, however, it is important to maintain a high level of control to maintain assay precision. Recent developments in automated workstations have resulted in capability to use an on-board four-place analytical balance to monitor the progress of the sample preparation process. This paper discussed several techniques built into the automated instrumentation which use analytical balance results to help ensure the precision and integrity of the analytical process. Some examples are:

(1) The use of concentrate weight to calculate and add diluent during automated dilutions.

(2) The use of sample weight to calculate and add a proportional amount of solvent.

(3) The use of sample weight to calculate and add a proportional amount of internal standard.

(4) The monitoring of the weight of all reagent additions to ensure adequate system performance.

---

### A robotized system for measuring and weighing specimens submitted for physical properties and chemical resistance testing

*R. D. Jones and J. B. Cross (Phillips Petroleum Company, Bartlesville, Oklahoma 74004, USA)*

Determining the physical properties of polymers and resins is a continuing effort at Phillips Petroleum Company. Among the parameters monitored are tensile strength, resistance to shear and compression, and the material's compatibility with various solvents.

Materials submitted for physical testing arrive molded or stamped in a variety of shapes. Before analysis can begin each specimen's thickness and width must be measured at selected locations. Samples intended for chemical resistance testing are also weighed.

Until recently this data was gathered manually, using a micrometer and a balance. But with sample volume running 30–40 000 specimens a year the task required five or more man hours per day. This paper described how a Zymate robot, commercially available hardware, and customized racks were used to automate the operation.

---

### CHEMICAL

### Robotic automation of a gravimetric determination for carbon and hydrogen

*Thomas R. Smith, Gregory J. Kamla, Gregory L. Rupert, Michael P. Kelly and Alan R. Forster (Shell Development Company, Houston, Texas, USA)*

The automation of a gravimetric combustion determination for carbon and hydrogen content of organic liquids was discussed. This system uses several Zymate II arms operating simultaneously from a single System V controller. The robot arms are used to load the samples into the combustion furnace, in addition to performing all the weighings of the gas absorbers used for the gravimetric determination. The robot also performs such tasks as capping and uncapping sample containers and pipetting of liquids as necessary to transfer the sample into the combustion furnace. The system can operate up to four combustion furnaces at the same time through interweaving of the required robotic manipulations. The System V controller and the combustion furnaces are operated as peripheral devices by an IBM compatible computer. All required sample information and the results of the determinations are handled by the IBM compatible computer.

The operator's involvement in the procedure is limited to placing sample containers in the sample rack and refilling the various consumables. All other phases of the analysis are computer controlled and are performed by the robots.

The robotic system is housed in a three-tiered custom robot enclosure that conveniently integrates the robot into the laboratory environment while maximizing efficiency and minimizing the amount of laboratory floor space required to perform the determinations.

### Robotic systems for analysis of photoprocessing solutions

*Kathryn L. Jansen, Faye M. Wilbur and Deborah A. Tabone (Eastman Kodak Company, Manufacturing Quality Assurance Organization, Rochester, New York 14650-1807, USA)*

Approximately 50 000 samples each year are received by the authors' quality control laboratory. This represents almost a quarter of a million individual chemical determinations. Internal clients, primarily from Eastman Kodak Company's Photographic Lines of Business, and from Marketing Technical Support, impact this sample load as new products are tested and developed. In addition to variations in number of samples submitted, changes to product formulations may necessitate moving an analysis to a different type of instrumentation or modifying existing analytical procedures.

Because of these concerns, any equipment brought into the laboratory has to be readily adaptable. On-line autosamplers are one way these needs have been answered. Implementation of laboratory robotic systems has allowed the lab to handle changing workloads, and it frees trained personnel to handle non-routine samples and interact more closely with their clients. Examples were given of applications based on Zymark and SFA (Source for Automation) robotic systems, illustrating custom features of these systems and gains achieved by conversion to robotic methods. The systems described include (1) preparation of samples for X-ray fluorescence measurements; (2) measurement of solution pH; and (3) sample preparation and determination of several analytes by titration.

### An adaptable robotic workcell for performing automated water determinations using Karl-Fischer titrimetry

*Brian D. Seiler (Eastman Chemical Company – Acid Division, Kingsport, Tennessee 37762, USA)*

*Lisa D. Whiteley (ICI Americas, Wilmington, Delaware 19897, USA)*

An industrial quality control laboratory at Eastman Chemical Company receives numerous samples for water determinations each day. Some of these represent product 'lots' and are submitted for 'specification' testing, but many samples are submitted as a part of an extensive statistical process control program. Samples continuously filter into the lab and water levels can conceivably range from 0–100%. Many different types of liquids must be tested, including non-routine samples that lab customers occasionally submit. An automation system designed to process large batches of similar samples (water levels falling in a rather specific, narrow range) is clearly not appropriate in this setting.

An adaptable, robot-based automation system has been designed to handle the special needs of this lab. Each sample is barcoded with all essential information related to the sample. In addition to identification information, values representing the anticipated water level and the number of replicate determinations desired are typically included. When this information is 'read' into the system controller, the sample size taken for each determination is automatically adjusted to compensate for the anticipated water level, and results are accumulated (if possible) until the specified number of replicates determinations have been performed. Statistical data is computed and aliquot-to-aliquot repeatability assessment can be performed during the course of routine operation.

A novel device featured in this system is a gravity-feed input rack. An optical sensor continually monitors the pickup position of the rack, and when the control program finishes processing a given sample, it loops back to 'see' if another sample is at the pickup point. Thus, the operator can place a sample at any time into the rack, at any queue position desired. The number of samples to be processed need never be entered into the controller. 'Priority' samples are promptly processed when they are placed at the front of the queue.

---

### PHARMACEUTICAL

### The evolution of robotics in a pharmaceutical quality assurance laboratory

*William P. Haller (Ortho Pharmaceutical Corporation, Raritan, New Jersey 08869, USA)*

Since the introduction of laboratory robotics in the early 1980s, the Quality Assurance Laboratory at Ortho Pharmaceutical Corporation has been taking advantage of the benefits this technology has to offer. This paper outlined why the Corporation decided to commit to laboratory robotics and how the systems were designed and implemented. The first system installed in the laboratory was programmed to perform a simple liquid/liquid extraction for content uniformity testing of oral contraceptive tablets to be analysed by GC. The system was soon expanded to also perform content uniformity and composite testing of oral contraceptives for HPLC analysis. Because this first system was dedicated to only testing oral contraceptive tablets, a second system was installed in the lab to prepare other solid dosage form products for both GC and HPLC analysis. This system was designed to be flexible so that many products could be prepared, as the volume of any one of these products would not be enough to keep the robot working all of the time. Recently, a third system was installed in the laboratory to perform dissolution testing of all of the Corporation's oral contraceptive tablets.

Besides set-up and programming of the systems (the first two systems were designed, set-up, and programmed by in-house personnel), one of the most important factors in successful implementation of robots is the validation of the systems. This paper also discussed the steps taken to validate and then periodically revalidate each of the systems.

### Robot validation for pharmaceutical product analysis—measuring success II

*Kevin J. Halloran, Ligaya M. Lagade and Dorothy E. Martynuk (Carter-Wallace, Inc., Wallace Laboratories Division, Research Services, Cranbury, New Jersey 08512, USA)*

Validation is a critical step in the analytical method development process. Now that firms are routinely delegating repetitive analyses to laboratory robots, robot validation has become a visible issue in the pharmaceutical industry. In this presentation, the authors discuss the strategy they adopted to ensure the reliability of our robotic applications. This discussion covered the various aspects of validation, including hardware, software and the overall system. Their criteria for evaluating the suitability of a method for automation was also explained. The highlighted application was the sample preparation of a pharmaceutical tablet dosage form for HPLC analysis.

### Revitalizing an older Zymate system for a dissolution/automation laboratory

*John Russell and Tara Boyd (Wyeth-Ayerst Laboratories, Rouses Point, New York 12979, USA)*

Laboratory automation is frequently designed for a specific assay or product, and the equipment is purchased to reflect the demands of the analysis. In this case, however, Zymate I equipment obtained from another division was upgraded to Zymate II; this 'new' robot formed the crux of a system designed to increase overall laboratory productivity and efficiency. This paper described the development of a dual HPLC injection system to accommodate a large number of dissolution samples. The robot arm now services two HPLC systems, more than doubling sample throughout, while maximizing use of available space. The advantages of the present system over an autosampler (for example a WISP) are discussed in terms of productivity, versatility, reliability and cost-effectiveness. Key points include:

(1) Maximizing the system design to increase overall laboratory productivity.

(2) Limitations of the existing system.

(3) Advantages of upgrading to System V technology.

### On-line robotics for multi-fermentor process monitoring and control

*K. D. Reda, M. P. Thien and R. L. Greasham (Merck, Sharp and Dohme Research Laboratories, Biochemical Process Research and Development, Rahway, New Jersey 07065, USA)*

A flexible laboratory robotics system was developed to provide on-line sample processing and analysis for up to six laboratory fermentors. The design combines a multi-fermentor sampling system, a Zymark robotics system, and a host HP1000 computer in a user friendly configuration. Logic was developed for the robotic controller to sample the fermentors at different sampling rates, to process samples as a function of the sampling source, and to process off-line samples or standards as time permits (i.e. on-line samples are given priority for periodic analysis). A user interface was designed to facilitate operation of the robotics system through the use of input switches. Processing variables are readily modified without interrupting the sampling schedule. Supplies such as reagent levels, pipette tips, and HPLC vials are monitored automatically to facilitate continuous operation. A wash station was developed for automation reuse of 50 ml tubes. Current sample processing capabilities include aseptic sample acquisition, sample weight measurement, chilled sample storage, solvent addition, capping/uncapping, vortexing, sonication, pipetting for secondary dilution, centrifugation, HPLC vial filling (i.e. through a septum), and/or HPLC injection. Sample processing information and chromatographic reports are transferred to the host computer for data processing, analysis and storage. On-line extraction and HPLC analysis of a secondary metabolite from dual fermentors has been implemented. Improved analytical precision over manual processing and analysis has been confirmed.

---

### MICROPLATES

### Clinical chemistry applications of robotic systems: cloning of a microplate management system and interface to an FPLC system for on-line compositional analysis of serum lipoproteins

*Dennis S. France, Robert E. Quinby, John Babiak, James R. Paterniti, Jr. and David B. Weinstein (Sandoz Research Institute, Department of Lipid and Lipoprotein Metabolism, East Hanover, New Jersey 07936, USA)*

The unattended quantitation of 17 different biochemical parameters by microplate assays using a Zymate II microplate management system has been previously described by the authors. Over the first two years of operation this system has performed over 250 000 analyses and compound-screening capabilities have been doubled. Data on system reliability and performance of assays over time were presented.

Analysis of lipoproteins isolated by ultracentrifugation is complicated by problems of quantitative recovery. Fast protein liquid chromatography (FPLC) provides excellent resolution and recovery of various lipoprotein classes, but possesses limited throughput when performed manually. With a growing reliance on robotic analyses the main functions of the initial system were duplicated and the capabilities of the second system expanded to include on-line analysis of lipoproteins separated by a FPLC column run by Waters HPLC system. The robot was interfaced to a Gilson FC203 fraction collector which collects 40 0·5-ml fractions in 96-tube microtubes for each serum sample injected. After each pair of injections and collection, the robot replaces the microtube rack on the fraction collector, and then performs designated microplate assays for cholesterol, phospholipid, triglyceride, and apolipoprotein assays. While column run-time (1·0 h) is still a limiting factor, the automation of this process has allowed a six-fold increase in the number of samples processed. The resulting data provides detailed compositional analysis of serum lipoprotein parameters in response to nutritional and pharmacologic perturbation. The software management of multiple top-level clinical chemistry programs has been significantly enhanced by the increased disk space and accelerated processing of the System V controller. Automation of routine biochemical analyses continue to provide high quantity and quality data, and to provide growth opportunities in other areas for bench scientists.

### Microplate technology: turning ideas into reality

*Jerry Hahn (Becton-Dickinson, Advanced Diagnostics Research Center, Baltimore, Maryland 21209, USA)*

Since the first automated microplate system was developed in 1984, the technology for automated analyses using microtiter plates has steadily evolved. The cost-effective use of microplate-configured assays in biotechnology, monoclonal antibody, and medical diagnostic services is now well established and encompasses additional hardware for mutiwell formats. This growth, from an idea to an effective analytical component in these laboratories, has been achieved through the collaborative efforts among vendors, industry, and most of all, system implementers and users. This paper highlighted many of the contributions these key people have made to the success of this innovative system. The current array of applications, some possible prospects for future development, and means of achieving these goals were reviewed.

### Application of robotics to the routine preparation of agricultural testing samples

*Judith Pelczar-Richmond (American Cyanamid, Agricultural Research Division, Princeton, New Jersey 08543, USA)*

In order to provide a more efficient and centralized means of preparation, a Zymate II Laboratory Robotics System was designed and installed for the automated preparation of acetone solutions of organic compounds for agricultural testing. This new system replaces the tedious and time-consuming method of manual sample preparation. Previously, a biologist would receive an entire testing sample circulated by the chemical library and weigh out an exact amount of dry material into a vial. The testing sample was then passed on to the next screening group and the procedure repeated. The Zymate II system uses a pre-tared test-tube which contains a rough-weighed sample. The compound net weight is calculated, acetone is added to achieve a specific concentration, the sample is homogenized and finally subsampled into four other vials and two 96-well microtiter plates. The solutions are then delivered to the biologists. This system handles a maximum of 40 samples per run.

All data generated by the Zymate II, such as the sample testing number, amount of compound in each vial and sample preparation date, is captured in ASCII file. The file is transferred to a VAX and the data is stored in S1032 and ORACLE databases for use by sample tracking programs.

### BIOLOGY/BIOTECHNOLOGY

### Robot automation to grow protein crystals for X-ray diffraction experiments

*M. A. Roos, A. F. Witt, J. R. Smith and R. A. Stegemen (Monsanto, St Louis, Missouri 63167, USA)*

One of the difficult tasks in protein X-ray crystallography is the ability to obtain large, well-ordered crystals, which diffract X-rays to high resolution. Classical manual methods of finding a set of conditions for crystallization of proteins requires the tedious preparation of hundreds of thousands of experiments.

The computer programs and automation developed at Monsanto greatly reduce the tedium of preparing these experiments. This paper described the software, robot, and associated work stations. Some of the automation's unique features are:

(1) Software to rapidly design multi-variable arrays of experiments.

(2) Multi-tasking computer control of hardware with interaction of data files and automation.

(3) Use of CRS-Plus robot with fixed axis articulation and interchangeable hands.

(4) Design of a multi-purpose Zymark hand with a vacuum pick-up, 25 μl syringe, and 500 microlitre syringe.

(5) The ability to seal between rectangular glass slides and tray compartments, by applying a bead of petroleum jelly with a precision of ± 0·005 in on a tray ridge 0·060 in wide.

(6) Optical detector to locate the X-Y-Z co-ordinate of a syringe needle tip, to the nearest 0·001 in. This information is used to correct any error in the robots tool transform, thus allowing precision placement of the needle.

(7) Preparation of hundreds to thousands of experiments each requiring a unique recipe to be dispensed from a library of 32 chemicals.

(8) Design of a self-cleaning mixer that also measures the pH of the mixture.

### Robot-tended crystal growth in a space-based laboratory

*Elaine M. Hinman (NASA, MSFC, Alabama 35812, USA)*

X-ray crystallography is one of the techniques used to understand the structure of proteins. This has led to the need to produce larger and better-formed crystals. One way to do this may be to grow the crystal in a microgravity environment, such as space. Since the first Spacelab Protein Crystal Growth experiment was run, significant interest in space-based crystal processing has been generated. Current space techniques have relied on human interaction with the growth chambers during the process. However, to expand on-orbit capabilities a higher level of laboratory automation will be required.

Automated laboratory concepts involving robotics have been developed. This paper discussed on-going work in the areas of ground-based crystal growth and handling using a robot, and microgravity verification of robot performance. The related areas of remote telescience and operator interface to the experiment were also touched upon. Preliminary results from these efforts were presented.

### Production of radioactive pharmaceuticals using the Zymark Robot

*J. H. Courter, J. M. Link, J. R. Grierson and K. A. Krohn (University of Washington, Imaging Research Laboratory RC-05, Seattle, Washington 98195, USA)*

The on-site production of radiopharmaceuticals with short half-lives for nuclear medicine involves working with small samples of very high levels of radioactivity, and a radiation field at contact of greater than 10 R/h. The manual production of these pharmaceuticals results in unacceptable radiation exposure to the chemists, and can involve human error. Automation reduces the exposure, resulting in a safer working environment and ensures consistent synthetic yields.

In the authors' research facility, 13 radiopharmaceuticals are produced routinely. Usually a particular synthesis is run only once in a day. In addition there is a space limitation as syntheses must be performed in a leadlined fume hood. Thus, it is not practical to have a dedicated synthesis setup for each product. An advantage of a robotics system is that it is modular. Only the stations required to meet particular needs must be installed.

Zymark has many stations useful in the radiochemistry lab, but some, which are tailored for repetitious tasks, are unsuitable for the authors' use. Stations have been built for handling single samples, but which include more than one function to save on space. Fortunately there are common chemical steps to different syntheses, such as retrieval and drying, trapping of volatile precursors, purification, and heating of reaction vessels to high temperature and/or pressure, so that many stations are usable in different syntheses. These custom stations and how they work with the Zymark standard pysections are illustrated by describing the syntheses that have been automated.

This work was supported by a National Institute of Health grants CA42045 and HL38736.

### The concept of modularity in the creation of automated analytical laboratory systems

*Gary W. Kramer and H. M. Kingston (National Institute of Standards and Technology, Center for Analytical Chemistry, Gaithersburg, Maryland 20899, USA)*

At the Center for Analytical Chemistry in the National Institute of Standards and Technology, a group of interested parties from the private sector and other government agencies have been gathered to form the Consortium on Automated Analytical Laboratory Systems (CAALS). The purpose of the Consortium is to foster the creation of automated chemical analysis systems which will improve the quality of analytical data, will facilitate the entire analytical process, and will promote the standardization and transfer of analytical methods while providing industry with competitive advantages in chemical measurement technology.

Most of the technology necessary to create systems for automating entire chemical analyses currently exists. However, interconnecting today's instruments to build such systems is often a difficult process. This stems in large part from the lack of general guidelines and standards in critical areas of data and control information interchange as well as sample manipulation. Application of the modular workcell concept, along with standardized intermodule communications methods, can dramatically ease the task of constructing automated analysis systems. Once constructed, automated chemical analysis systems will prove to be a valuable tool for achieving and maintaining quality assurance in the laboratory.

### MANAGING LABORATORY AUTOMATION

### Robotic applications: lessons on what constitutes success

*Beth Hutchins (Genentech, Department of Medicinal and Analytical Chemistry, Biological Chemistry Section, South San Francisco, California 94539, USA)*

At Genentech varying degrees of success in applying robotics to laboratory situations have been encountered. The fiscal analysis of robotics is promising, particularly in a climate where manpower growth is limited. The ability of a robotic system to reliably take over tedious tasks has been demonstrated. Yet implementation and successful utilization of robotic applications is not based on the ability to design and program the instrumentation. Successful implementations require education, careful planning of the installation process, and persuasion.

There are a number of robotic applications at Genentech. There are several that are perceived as obvious success. One 'success story' is the application of robotics to automating the iodinations of proteins and peptides by a variety of chemistries. The success of this operation lies in the fact that the system is dedicated to a defined application, that it enables laboratory personnel an added measure of safety in reducing their exposure to gamma radiation, and that it was implemented in the department in a short time-frame while still meeting the expectations agreed upon. Additionally, an open-house demonstrated the measure of its success; now other departments want similar systems of their own.

Experiences with microplate management systems provide additional insight into what constitutes successful implementation. In Genentech's research and development laboratories most experiments require flexibility. Change is the name of the game. One robotic application offers a variety of methodologies and has a program which is easily modified for new methods. Because of its capabilities, it could be labelled successful. In practice, however, this robotic system is used infrequently. A second microplate system which screens samples in any of five different assays is used more frequently. Here, there is a need to screen a large number of samples weekly, even though any or all of the five different assays may be required for the screening. The fact that this is a 'dedicated' system is the major reason that it is successful, even though it too was designed with flexibility built into it. The lesson here is the necessity of fine-tuning the application and carefully defining the objective of the project prior to its implementation.

### Managing robotics in the bioanalytical/metabolic environment of a pharmaceutical company

*Stanley Kushinsky (Syntex Research, Palo Alto, California 94304, USA)*

The establishment of a successful robotic unit within Syntex Research's facilities involved a major commitment of time and effort. Among the required actions were: (1) learning what operations a robotic system can do efficiently (for example, through published literature as well, as through input from vendors and users of relevant commercially available systems); (2) assessing whether or not the capabilities of a robotic system are consistent with ongoing or projected activities; (3) becoming convinced that having a robotic system would be beneficial; (4) deciding which procedures would be most suited to performance robotically; (5) obtaining cost estimates for the required equipment, evaluating the alternatives and selecting the most appropriate system; (6) locating adequate space for the robotic system and obtaining estimates of the cost of any required modifications to facilities; (7) preparing an appropriate justification for capital funding; and (8) selecting suitable personnel to participate in the planning, implementation and subsequent operation of the system (listed last, but, unquestionably, the most critical factor).

The resulting system (Zymark Zymate II PyTechnology, described in detail by G. Lee, K. Hama and I. Massey at this symposium) includes the following features: liquid-liquid extraction or solid phase extraction, weighing station, centrifugation, solvent evaporation, fully automated HPLC with signal output to a centralized data acquisition/laboratory information management system or with selective collection of fractions for subsequent RIA. Evaluation of performance included: (*a*) precision, accuracy and reproducibility of the results in comparison with those obtained manually; (*b*) reliability; (*c*) comparison of manual and robotic throughputs; and (*d*) cost-benefit analysis. This evaluation led to the conclusion that performance of the initial system greatly exceeded expectations and funding for expansion of the robotic facility currently is being sought. The analytical procedures employed thus far involve relatively straightforward adaptations of existing manual methods but with greatly facilitated control of quality. With optimal use of robotics, it is possible to accommodate significantly increased workloads without any increase in manpower, while providing the operators with much greater opportunities for evaluation of data, improvement of methods and development of new strategies. The opportunities for improvements often are limited only by the ingenuity and innovativeness of the operators. A potential problem that remains to be addressed is employee anxiety over possible changes in the size and character of the work force if the use of robotics expands dramatically.

### Challenges facing modern automated laboratories

*Elsayed A. Elnenaey, Josip Pluscec and Vincent Fernandez (Bristol-Myers Squibb, Technical Operations, US Quality Control/Quality Assurance, New Brunswick, New Jersey 08903, USA)*

The recent trend in the wide variety and large number of samples to be processed daily by analytical laboratories has put tremendous burden on managers and employees of these laboratories. Analytical instruments have been automated to run 24 hours/day. However, the most labour-intensive step in chemical analysis still remains 'manual sample preparation'.

Recent developments in laboratory robotics have made it easier to perform difficult tasks in sample preparation. These include dissolution testing, content uniformity, assays, microbial contamination testing, handling biological samples, process control, fermentation, environmental etc.

Laboratory managers will have to face the new challenges by preparing their laboratories, and training personnel for the advanced robotic technology. The presentation addressed other issues such as planning, cost analysis, specific applications installation, validation and final approval for testing.

Certain guidelines regarding physical space, cost-effectiveness, utility services, robotic friendly methods, multi-applications, training and maintenance were discussed.

### Managing an automation development group

*S. D. Hamilton (Lilly Research Laboratories, Lilly Corporate Center, Indianapolis, Indiana 46285, USA)*

Automation is one of today's key issues in the analytical laboratory. We are inundated by instrument manufacturer's promises to solve our problems with their latest state-of-the-art equipment. Laboratory managers are eager to find solutions to the dilemma of an ever increasing workload and constraints on staffing. Scientists anticipate breaking new ground with more advanced instrumentation. The question arises: What automation is right for me and how do I get the most out of the investment?

Answering this question requires a careful examination of the laboratory situation. What a given laboratory needs, can afford or can support depends on factors such as facilities, laboratory and support personnel, customer needs, corporate priorities, training, funding and equipment. The role of an automation development group is to develop an automation strategy based on an examination of all these factors rather than on an impulse to 'keep up with the lab next door'.

This paper examined crucial issues, while drawing from experiences within Lilly Research. Current trends in our automation philosophy and technology were explored and some projections for the future offered.

### Six years of robots

*G. F. Plummer (ICI Pharmaceuticals, Macclesfield, UK)*

A Zymark laboratory robot was first introduced into ICI Pharmaceuticals in October 1984, for use within the Drug Kinetics Group of the Safety of Medicines Department. The purpose of this system was to automate analytical methods for the determination of drugs in biological fluids, using liquid/liquid extraction. Since then numerous analytical procedures for ICI compounds have been successfully applied to this form of automation and the number of robots used within the group has increased to five, with an additional robot also in use elsewhere within the department. These robots have been purchased as hardware only systems and all the configuration, programming and the serialization of applications has been carried out in house, along with the design and manufacture of special modules.

Upon purchase of the second robot in 1986, a policy of cloning the systems and developing 'generic' and multifunctional analytical robots was adopted. The purpose of this approach being that all present or future 'drug analysing' robots would be able to perform any of the automated analytical methods. Although this approach does lead to a high degree of versatility, and subsequent usage of the individual systems, it does, however, promote its own set of problems.

The 'drug analysing' robots all have the capability to isolate drugs from the biological fluids by liquid/liquid or solid phase isolation techniques, either of which can be interfaced to gas or liquid chromatography systems. Procedures vary from simple single forward extractions to complex preparations involving low volume hexane/water or methanol/water/chloroform partitions, in addition to initial forward extractions, as clean ups prior to enantiomer selective chromatography. There are also procedures which pre-extract plasma samples for RIA or ELISA.

Since the initial analytical procedure went 'live' in 1986 the annual sample throughput has rapidly increased from about 4–5000 assays in that year, to 28 000 in 1989, with initial figures 1990 indicating a possible turnover of around 33 000 assays.

Although the major robotic effort has been directed towards drug analysis, for the generation of pharmacokinetic data, additional systems have been set up within the Metabolism group and the Departmental Dispensary. The Metabolism robot has been successfully applied to the automation of a Packard 306 Oxidizer and as such is our first PyTechnology robot. The Dispensary robot was set up to automate the preparation of medical vials containing an antibiotic material, involving the addition of sterile water to the vials and the subsequent mixing of the contents to ensure dissolution. This system will later be reconfigured to perform diet analysis.

Consequently, this paper endeavoured to convey the author's experiences over the past six years with Zymate robots and their impact within this Drug Kinetics Group.

### Automated sample preparation: challenges and opportunities

*W. J. Hurst (Hershey Foods, Hershey, Pennsylvania 17033, USA)*

Since the introduction of laboratory robotics in the early 1980s, one of its prime uses has been automated sample preparation. While automated sample preparation in its various forms has greatly impacted laboratory operations and productivity, it is not without its own set of limitations. These limitations range from laboratory constraints to personnel issues. This presentation discussed a number of these issues that are important in this area and recommended ways to eliminate or reduce these potential problems.

### ENVIRONMENTAL

### Automation of biochemical oxygen demand (BOD) in waste-water

*W. A. Michalik, R. M. Zerkel and J. B. Maynard (Shell Oil Company, Wood River, Illinois 62095, USA)*

The procedure for determination of the biochemical oxygen demand (BOD) of waste-water given in *Standard Methods* 16th Edition (1985) has been automated with a Zymark Laboratory Automation System using a custom BOD workstation. The BOD workstation does all glassware and sample manipulations, except movement of the filled BOD bottles to and from the incubator. The system handles all sample transfers, as well as the additions of dilution water, biological seed and phosphate buffer. Dissolved oxygen readings are made before and after incubation with a YSI Dissolved Oxygen Meter. The sample transfers and additions of dilution water are made with calibrated peristaltic pumps rather than using conventional pipettes. The robotics system also contains a workstation to wash and rinse the BOD bottles after use.

The precision of the BOD results obtained with the robotics system has been equal to that obtained using the manual BOD procedure. Further, automation of the BOD analysis has significantly improved laboratory productivity by reducing analyst involvement in BOD testing by 75%.

### Robotic automation of gravimetric methods used to test environmental samples

*L. W. Lindquist and F. X. Dias (WMI Environmental Monitoring Laboratories, Inc., Geneva, Illinois 60134, USA)*

Environmental samples are analysed for total dissolved solids (TDS) dried at 180 °C, TDS dried at 103–105 °C, total suspended solids (TSS) dried at 103–105 °C and total solids (TS) dried at 103–105 °C using approved Environmental Protection Agency (EPA) methods. These gravimetric methods are rather labour intensive and some require vacuum filtration of the water samples that are processed.

A Zymark PyTechnology system has been designed to automate these four methods. A TurboVap has been incorporated into the design of the system to speed the evaporation of the samples. The paper provided an outline of the system and procedure used, as well as an estimated system payback period.

### Robotics applications in an industrial environmental laboratory

*Walter Anderson (Environmental Services, Inland Steel Company, East Chicago, Illinois 46312, USA)*

The robotics application at the authors' environmental laboratory has been set up to analyse pH, biochemical oxygen demand, and suspended solids using the Zymate II robot. These tests were selected for automation because they are tedious to perform and have simple chemistries. However, even the automation of simple chemistries can be subject to unexpected events that would require stopping the robot to make corrections and then continuing with the analysis.

A sophisticated interface was developed to allow the operators to stop and restart the robot at any moment during the analysis of these tests – without the use of recovery programs. This interface allows the robot to autostart itself in case of power interruption and continue with the analysis. In addition, an operator can reanalyse a sample while any of the test programs are running. The system also permits the robot to pause during an error condition and request operator assistance.

The automated chemistries were described, along with the interface for error detection and restarting.

### Automation of EPA test methods for groundwater samples: sample preparation for mercury analysis and suspended solids analysis

*R. K. Bergman, R. C. Elmer, K. A. Hendrickson and L. R. Lofgreen (Hercules Aerospace, Magna, Utah 84044, USA)*

Sampling testing to ensure compliance with EPA requirements is very labour intensive due to the number of samples, number of tests, and type of analyses required. In one year, over 500 groundwater samples will be taken at the authors' plant, requiring testing for up to 50 different parameters. Approximately 18 000 analyses will be performed. While the amount of work increasing, there has been no increase in the number of personnel available to perform the work. Automation of some of these tasks will assist the laboratory in processing the growing sample load. This paper discussed automation of sample preparation for mercury analysis and automation of the suspended solids analysis.

Sample preparation for mercury analysis (EPA Method 7470) involves over half of the time required for this test. From an automation viewpoint, the primary difficulty with this test is the large number of samples, in large (300 ml BOD bottles) vessels, which must be processed. By combining batch and sequential programming techniques with a special 'racetrack' sample handling rack, a system has been developed which appears to be a simple two position rack to the robot, but enables it to process up to 48 samples in a batch. This has resulted in a labour reduction of 85% for sample preparation; a 50% reduction in labour for the total analysis. Efforts to automate sample analysis by the cold vapour technique were also discussed.

The suspended solids analysis (EPA Method 160.2) consists of several operations which are quite complex for a robot to perform, making this a very difficult test to automate. A discussion of the custom equipment developed for this operation will include an automated vacuum filtration funnel assembly, robot friendly desiccator and filter rack, a compact version of the 'racetrack' sample rack, and a vacuum pickup device for handling filters so that sample material is not disturbed.

### CHEMICAL

### Automation of 11 distillation stills for the determination of nitrogen-containing impurities in hexamethylenediamine, adipic acid, and their salt, Part I

*Darrell A. Kelly and James D. Cunningham (Monsanto, Pensaçola, Florida 32575, USA)*

Attempts to automate or change the method of analysis to determine the reducible and hydrolysable nitrogen compounds in hexamethylenediamine, adipic acid and nylon salt have not been successful until the advent of the Zymark robot system. Two robot systems now carry out the analysis with minimum technician involvement. The reasoning and philosophy involved in the project was discussed and a typical analysis described. Technician time has been reduced from approximately 1–2 h down to 5–10 minutes per sample. Statistical analysis of the data indicate the automated system is comparable to the older manual method. The projected yearly return on investment of 75% versus actual savings appears to be conservative based on actual laboratory charges.

### Automation of 11 distillation stills for the determination of nitrogen-containing impurities in hexamethylenediamine, adipic acid, and their salt. Part II

*James D. Cunningham and Darrell A. Kelly (Monsanto, Pensacola, Florida 32575, USA)*

Automation of 11 distillation systems for the analysis of nitrogen-containing contaminates in hexamethylenediamine, adipic acid, and hexamethylenediamine adipate (nylon 66 salt) was accomplished using two Zymark Zymate II robot systems. The procedure is applicable to the determination of ammonia - aminocapronitrile, and hexamethyleneimine in hexamethylenediamine and nitrogenous compounds in adipic acid or nylon salt, that are converted to ammonia and distilled off in methanol and/or water.

Each Zymark Robot monitors five or six stills with thermistors and level indicators, adds methanol or water solvents and concentrated NaOH reagent. Under program control, from two to eight cuts of 50 ml are made per still. Cuts of three to one ratio are taken using a magnetic separator controlled by the PECs. Samples from MARGE (Monsanto's Analytical Robot Group Endeavor) are titrated on a first-priority basis, those off LARS (Laboratory Analytical Robot System) on second priority, and hexamethylenediamine concentration assay on a third-level priority.

Sample cuts are passed between robots using a rack with switch interfacing to communicate readiness. Titrations are done on a Brinkmann 682 Metrohm Titroprocessor with two 665 Dosimats.

System hardware consists of two Zymark II Robots (with CPU3 processors) and two controllers, nine Power and Event Controllers, two Liquid Handling and Event Controllers, two Meanwhile cards, one Brinkmann Titroprocessor 682 with two 665 Dosimats, two cup dispensers, a Mettler AE163 Balance, six masterflex peristaltic pumps (four heads each), two HP Thinkjet Printers, six magnetic separators, 11 heating mantels, over 30 solenoids, 11 thermistors, six liquid level detectors, and two photodetectors.

### Ten years of search for excellency in obtaining analytical data

*Jean Petin (Laborlux S.A., Esch-sur-Alzette, Luxembourg)*

Laborlux Laboratories are an independent organization working mainly for the steel industry. The biggest work-load is elemental analysis using XRF, ICP-OES, ICP-MS, AA, and F-AA, but ion chromatography for anions and GC-MS or FF-IR for organics, mainly emulsion oils and transformer oils are also used. Besides materials control, environmental control is taking a permanently increasing work-load.

Twelve years ago, the materials oriented wet chemist was the expert and instrumental production control methods were calibrated against his results. Colorimetric methods and atomic absorption were the only instrumental methods used in the author's central laboratory environment. ICP-OES in 1979, and XRF combined with fused bead sample preparation techniques in 1981, backed up by home-made LIMS system, changed the laboratory progressively into an analysis factory. Analysis was no longer material oriented, but instrument oriented. Sample preparation, measurement, validation of results on a set of samples became the job of different people. Calibrations are set up against synthetic standards and verified by certified reference materials.

From 1984, Zymark robotic systems started to take over an ever increasing work-load on sample preparation. Fused bead preparation for X-ray and acid dissolution of steel-samples were automated. Most instruments are now equipped with autosamplers. Microwave dissolution techniques have been adopted and will soon be automated. Individual workstations simplify and standardize remaining manual operations. This approach significantly improved the chemist's working environment. The analysis factory concept had transformed the craftsman he was into an assembly line worker; now, he is becoming progressively an organizer and supervisor.

In order to improve the tools for this new kind of chemist, the author's company implemented in 1989 a more powerful LIMS-system called CCLAS developed by an Australian company. This system allows the easy connection of all instruments and has powerful quality assurance features. LIMS, automated instruments, robotic sample preparation are the elements making it possible to create a system giving the right numbers and showing at any time how these numbers have been obtained (audit trail).

To arrive at this stage people must understand the philosophy and participate to put into practice all the requirements to satisfy quality assurance regulations and ISO 9000 standards. The reward is a motivating and pleasant working environment.

### Process monitoring of polyethylene filler level using robotics

*Terry L. Combs (Quantum Chemical Corporation – USI Division, Rolling Meadows, Illinois 60008, USA); and Thomas Kinzelman (Zymark Corporation, Hopkinton, Massachusetts 01748, USA)*

The use of robotics has historically been restricted to either manufacturing or to laboratory use, seldom has laboratory robotics been used to monitor a manufacturing process. This paper described the use of a robotic test that monitors the percentage filler level in the production of compounded polyethylene.

The test historically used required a minimum an hour of analysis time and was not responsive enough to give sufficient control of a manufacturing process. A new test needed to be developed that would lend itself to providing a quick automated test and still preserve the integrity of the original test. Old chemistries were challenged and a test was developed that gave equal, if not better, accuracy and precision.

The new test method had to be automated to the extent of being capable of performing the test unattended for a minimum of 8 h at a time. The Zymark robot and PyTechnology were chosen to perform the test because of the ease of learning the programming and routine opeation of the equipment. The ultimate home for the system was to be a remote location, which necessitated a robot that was simple to use and understand, but which was capable of reliable perrformance in carrying out an analytical test.

The automated test had to be implemented into the manufacturing process using a sampling system on a side stream of the pelletizing operation that would provide a test sample for the robot upon demand. Finally, the real-time test results had to be displayed at the unit in the form of a control chart, using Lotus 1-2-3, to highlight trends and relative position of the sample in the product specification.

### Improved use of ISE's through robotics

*Bernard D. Stabley (Allied-Signal Corporation, Petersburg, Virginia 23804, USA)*

The analysis for fluorine on nylon fibres has been performed for years according to the method prescribed by the manufacturer of the fluorine sensitive Ion Specific Electrode (ISE). The method is not linear to low concentrations and is greatly technique dependent. Efforts to improve the accuracy of the method were successful, but greatly lengthened analytical time. The change to robotics not only improved the speed of analysis, but also improved precision and gave insight into ways to extend the linearity using an ISE into concentrations which were previously not possible.

### Robotic automation in the analysis of aflatoxins

*L. Pieta (Best Foods, Union, New Jersey 07083, USA)*

Robotic automation has vastly changed the previously tedious preparation of peanut-butter and peanut products for the determination of aflatoxins. Not only is the robotic procedure more efficient, but it provides better precision and reduces the handing of aflaxtoxin-containing materials by the analysts. Sample preparation includes clean-up with an affinity column and injection into a reversed-phase HPLC with post-column iodine derivatization and fluorescence detection. The accuracy, precision and recovery data were presented, as well as an analysis of cost savings.

---

### BIOLOGY/BIOTECHNOLOGY II

### A fully automated robotic system for the radioimmunoassay of steroid and peptide hormones using 'coated tube' and 'coated bead' techniques in an endocrine research laboratory

*R. H. Underwood, G. R. Bradwin and G. H. Williams (Brigham & Women's Hospital, Harvard Medical School, Boston, Massachusetts 02115, USA)*

For the last two decades or more, radioimmunoassay has been, and still is, the major technique used for assaying hormones in biological fluids, particularly human plasma. The evolution of solid phase supported antibodies, such as those immobilized in 'coated tube' and 'coated bead' techniques, has culminated into methods which could potentially be automated. A robotic system, employing the Zymate II, has been designed to fully automate radioimmunoassay methods employing these techniques and has been used for the assay of steroid and peptide hormones in plasma and adrenal cell preparations. There was a significant correlation ($p < 0{\cdot}05$) between the results obtained on this system and those obtained by a manual assay. All samples were run in duplicate and the precision and accuracy were considerably higher on the robotic than on the manual assay.

### Automation of radioimmunoassays

*Gail Freeman, Joy Gales, Diane Mayer, Faith Shulruff and Charles Jeuell (Searle & Company, Skokie, Illinois 60077, USA)*

Procedures for two radioimmunoassays (RIAs), prostaglandin $E_2$ ($PGE_2$) and cyclic AMP (cAMP), were developed using the Beckman BIOMEK 1000Automated laboratory workstation for routine analysis. Automation was accomplished by the implementation of several labware and procedure modifications. The goals were to eliminate the repetitive and tedious manipulations of manual assays and to increase analytical throughput. Performance characteristics of manual and automated methods were compared by evaluation of precision (coefficient of variation, %CV) and accuracy (analytical recovery, %AR). The goals were achieved; however, automation did not result in improved performance characteristics. An analysis of the manual and automated versions of the methods was presented in order to identify the components of the automated methods which need to be addressed to improve performance characteristics.

### Pharmacology, a new application for robotics

*P. Masson, T. R. Jones, E. Champion and L. Charette (Merck Frosst Center for Therapeutic Research, Pointe Claire-Dorval, Quebec, Canada)*

The use of robotics to carry out physiological, chemical and biochemical tasks is becoming more and more prevalent in both industrial and academic settings. The present paper described a novel application of a Zymate II robot in an industrial pharmacology laboratory. The procedure involves using a robot to administer serial dilutions of leukotriene $D_4$ ($LTD_4$) to a bank of eight tissue-baths containing guinea-pig tracheal smooth muscle. Firstly, the robot prepares two-fold serial dilutions (2048 ng/ml to 0.25 ng/ml of the agonist ($LTD_4$). Secondly, at predetermined time intervals, the robot dispenses, in sequence, 10 l of each dilution to each of the eight tissue baths. The tissues subsequently generate incremental increases in tension in response to cumulative addition of the agonist under study. A complete dose response curve can then be generated. Tissue contractions are recorded simultaneously on a Bechman Type-R Dynograph and on a PC using data acquisition software (Branch Technology). The robot also controls the washing cycle for the tissues which allows recovery from the first dose response curve. A second dose response curve is obtained following the recovery period. The manoeuvres are routinely executed with accuracy and reproducibility. The control EC50s (effective concentration producing 50% of the maximum contraction) vary less than 1% within any given experiment. This is well within the limits of variation achieved with a manually performed experiment.

This system will carry out an 8 h to 9 h experiment with a minimum of human intervention. The net result is a significant increase in drug testing capabilities.

### Validation of binding methods for epidermal growth factor and muscarinic receptors performed by a Zymate II robot for use in high volume screens

*S. L. Myers, G. Hingorani, L. Lauffer, D. Fry, L. L. Coughenour, and C. Clark (Parke-Davis Pharmaceutical Research Division, Warner Lambert Company, Ann Arbor, Michigan 48106, USA)*

In the pharmaceutical industry, an important source for new chemical leads is high throughput screening of large existing compound banks in receptor binding assays. The routine and repetitive nature of radioligand binding screens make them suited for adaptation to robotics technology. In this study two receptor binding methods performed by a Zymate II robotics system have been validated. One assay, for epidermal growth factor (EGF) receptors, used microtiter plate applications to assess binding to formaldehyde-fixed cultured A431 cells. The other method was a filtration assay for the binding of agonists and antagonists to muscarinic receptors in rat cerebral cortex membranes using a minitube format. Binding methods were executed by the Zymate II robot in batch and serial manner. Inhibition of [125-I] EGF by unlabeled EGF revealed no difference in IC50 values determined by manual or robotic procedures. Competition curves for arecoline and atropine against either [3H]-quinuclidynl benzilate (QNB) or [3H]-cismethyl-dioxolane (CMD) labeled cortical membranes were essentially the same for manual and robotic determinations. For each data point in the binding competition curves, between assay variability (SEM) was less than 15% of the mean percent inhibition with both manual and robotics methods suggesting that designation of a test compound as active or inactive from one-concentration binding screens would be equally reliable by either method. The Zymate II system was versatile in that it could be used for both cultured cell and membrane binding assays. Filtration of membrane samples and the preparation of either breakaway plates or filters for scintillation counting of receptor bound radioactivity were carried out manually. The use of a robotic system for sample preparation in high volume binding screens is advantageous, since, unlike humans, its pipetting accuracy is not compromised by fatigue and boredom. The development of filtration radioligand counting systems which can be integrated into the Zymate II robotics system should make its use for high volume binding screens even more cost efficient.

---

### DRUG METABOLISM

### The semi-automated extraction of captopril in biological fluids with a Zymark BenchMate for analysis by GC/MS

*Mark E. Arnold, Clifford J. Sachs, Eugene Ivashkiv, Mohammed Jemal and Allen I. Cohen (Bristol-Myers Squibb Pharmaceutical Research Institute, New Brunswick, New Jersey 08903, USA)*

The extraction methods for free captopril in urine and total captopril in plasma and urine have been automated using a Zymark BenchMate. Development of the methods was on a beta test unit undergoing evaluation in the authors' laboratory. The evaluation afforded the opportunity to use the versatile system prior to general market release and thereby increase the productivity of these captopril extraction procedures. Some of the beta test findings resulting in enhanced product development were reviewed. The chemistry of the manual methods for solid phase extraction of free and total captopril was described, as well as the BenchMate's program and method of sample processing. Typical GC/MS chromatograms for manual and automated methods were compared. Validation results from the GC/MS were presented.

### Automated sample preparation of serum cotinine for GC/MS analysis

*D. B. Shealy, R. H. Hill, Jr., D. L. Orti, S. L. Bailey, B. B. Miller and W. E. Turner (Centers for Disease Control, US Department of Health and Human Services, Atlanta, Georgia 30333, USA)*

One of the most important public health problems today is smoking. Exposure to tobacco smoke may be direct or indirect (passive) and can be difficult to evaluate particularly the passive route. Cotinine, a metabolite of nicotine, can be measured in serum to indicate exposure to tobacco smoke. An automated sample preparation was developed for the determination of cotinine in serum using gas chromatography/mass spectrometry. The presentation described the use of laboratory robotics for this determination and provided preliminary information about experience with this procedure.

### A PyTechnology robotic system for the analysis of xenobiotics in biological fluids

*G. J.-L. Lee, K. Hama and I. J. Massey (Department of Bioanalytics and Mass Spectrometry, Syntex Research, Palo Alto, California 94304, USA)*

An important part of drug development is the definition of the pharmacokinetics of the drug and/or its metabolites. This requires the development of analytical methods for the quantification of the drug and its metabolites in complex biological matrices such as plasma and uriune. The methods, once developed, are typically used for the analysis of large numbers of samples from pharmacokinetic, bioequivalence, clinical efficacy and toxicological studies. The routine performance of these methods is well suited for automation by a robotic system.

A Zymark PyTechnology Robotic System was set up to automate some of Syntex Research's analyses. The robotic system includes the following features/capabilities, liquid – liquid extraction or solid-phase extraction; weighing station, sample refrigeration, centrifugation; solvent evaporation; HPLC injector; fully automated HPLC with signal output to a centralized data acquisition/data processing/laboratory information management system or with selective collection of fractions for subsequent RIA. To date, the system has been set up to automate three methods. Method 1 employs protein precipitation and quantification by HPLC analysis with fluorescence detection. Method 2 involves solid-phase extraction and quantification by HPLC analysis with UV detection. Method 3 involves liquid – liquid extraction and HPLC purification of the extract, collection of the HPLC effluent containing the analytes of interest, and off-line RIA of the analytes. Implementation of Methods 1 and 3 invlolved simple modification of previously developed manual methods, whereas Method 2 was specifically developed for the robotic system since the manual method previously developed involved several liquid-liquid extraction procedures that could not be performed efficiently using the robot. Although substantial additional effort was needed for development and validation of the modified method, nearly all the necessary work was performed automatically by means of the robotic system. The robotic system has been set up so that any of the three methods can be performed simply by providing the computer with the appropriate method file name; no physical alterations need to be made to the system. As such, the robotic system can be used by non-robotic experts for the analysis of samples by these methods. The accuracy and reproducibility of the robotic methods equals or exceeds that of the manual methods. The system is very reliable and sample throughput of the robot on a weekly basis exceeds that of a chemist.

---

### DRUGS OF ABUSE

### Quantitative analysis of heroin samples by laboratory robotics

*Robin J. Ridgeway (Forensic Chemist, Drug Enforcement Administration, Washington, D.C. 20532)*

Heroin exhibits are frequently encountered by the DEA Mid-Atlantic Laboratory. An automated procedure has been developed to prepare and quantitate these exhibits, which allow for analyses to run unattended over a 24-hour period. System configuration consists of a Zymark Zymate II Laboratory Robot interfaced to a Hewlett-Packard Model 5890 Gas Chromatograph with a HP3396 Integrator.

Approximately four-fifths of the heroin exhibits range in potency from 3%–30%, with the others being as high as 90%. This wide potency range necessitated the writing of two programs to avoid overloading the GC column. The programs are written to allow for operation by chemists who are not specialists in laboratory robotics.

The automated system performs the analyses utilizing the following procedure: calibrating the authenticated drug standard, obtaining sample weights, dispensing internal standard solutions, filtering, transferring a portion to a GC vial, placing the vial into an autoinjector, and initiating the GC quantitative run. Liquid dispensing, pipetting, and weighing processes were validated. GC quantitative method is an adaptation of a validated manual procedure in general use throughout the DEA laboratory system.

### Automated analysis for cocaine in case samples

*Robert W. Taylor and Sam D. Le (Los Angeles County Sheriff's Department, Los Angeles, California 90057, USA)*

A totally automated procedure using the Zymate Laboratory Automation System for the extraction and derivatization of cocaine and benzoylecgonine (cocaine metabolite) from urine case samples was presented. Using a Zymate II Robot along with a Zymark custom liquid/solid extraction PySection for use with Detectabuse solid phase extraction columns, cocaine and benzoylecgonine are extracted from urine case samples. The benzoylecgonine is then derivatized to its trimethylsilyl ester. The underivatized cocaine and derivatized benzoylecgonine are detected by electron impact mass spectrometry in the selected ion monitoring mode. Both cocaine and benzoylecgonine are detected at concentrations of 50 ng/ml. In addition, the types of cases that are analysed at the crime laboratory and some of the outcomes of these cases were discussed.

### Rapid and efficient robotic extraction of cocaine and its metabolite from blood and urine by solid phase chemistry

*Bruce Houlihan (Forensic Science Services, Orange County Sheriff-Coroner Office, Santa Ana, California 92702, USA)*

Yearly increases in case load at the Orange County Sheriff Forensic Laboratory in California created a need for more rapid and efficient extractions methods for drug abuse testing.

Robotics was chosen as the most viable solution, primarily because of its ability to perform routine, repetitive tasks efficiently. Furthermore, the robot frees a significant amount of analyst time which can be used for more thought-intensive processes. The majority of crime-related toxicological samples in (blood or urine) screening positive for drugs of abuse in Orange County contain cocaine and/or its primary biological metabolite, benzoylecgonine. The cocaine extraction was chosen for adaptation to robotics for this reason. Initially, the robotic cocaine extraction was designed to mimic the manual liquid – liquid extraction performed by laboratory analysts.

The liquid–liquid extraction method was subsequently replaced by solid phase chemistry, however, which allowed for more consistency among samples, thus creating a predictable series of steps for the robot to perform. With the exception of drawing an initial aliquot of blood, the entire extraction procedure is performed by the robot prior to confirmation and identification by GC/MS. The analyst is only required to derivatize the sample, and then set up an automated GC/MS analysis. Current method development is an extension to the robotics now in use, where the robotic system will free up more of the analyst's time by carrying the sample through the derivatization and GC/MS sample preparation steps, and inject the sample directly.

---

### DATA MANAGEMENT

### Data acquisition between a Zymate system and the HP 3350A LDAS system

*John Garner (R. W. Johnson Pharmaceutical Research Institute, Raritan, New Jersey 08869, USA)*

This paper detailed the procedure for interfacing the Zymate robot to the HP3350A laboratory automation system for the transfer of sample weight data. Emphasis was placed upon the requirements and protocol of the Zymate robot to establish communication to any host computer system. The discussion and implementation of the interface were presented from the perspective of achieving total automation for an analytical method.

### Robotic sample preparation and HPLC analysis of lyophilized salt and liquid injectables

*Edward G. Kanczewski, Robert Doerig, Marie Skrilec and Robert E. Daly (Parke-Davis, Warner Lambert Company, Morris Plains, New Jersey 07950, USA)*

Automated sample preparation of lyophilized salt and liquid injectable dosage forms is performed on vials of different volumes. Lyophilized salts are initially reconstituted while liquid formulations already are in solution. Subsequent processing includes serial dilutions in order to prepare for HPLC analysis. Product specific variables are incorporated into the analysis by utilizing a product template. Multiple products are analysed on this system without significant modification of hardware or software. HPLC analysis is accomplished in conjunction with an in-house LIMS system. Post-analysis activities include: column washing followed by HPLC shutdown and the washing of test tubes used in the preceding determination.

### Automated intelligent control of microwave sample preparation

*P. J. Walter,[1,3] H. M. Kingstone,[2] F. A. Settle,[4] M. A. Pleva,[5] W. Buote,[6] J. Christo[6] and T. C. O'Haver.[3] ([1] Department of Chemistry, University of Maryland, College Park, Maryland; [2] National Institute of Standards and Technology (NIST), Center for Analytical Chemistry, Inorganic Analytical Research Division, Gaithersburg, Maryland; [3] Guest Scientist at NIST; [4] Department of Chemistry, Virginia Military Institute, Lexington, Virginia; [5] Department of Chemistry, Washington and Lee, Lexington, Virginia; [6] Zymark Corporation, Hopkinton, Massachusetts, USA)*

Microwave sample preparation is gaining support within the analytical community as a useful dissolution technique. Moreover, because of its physical chemical characteristics, it is becoming an ideal method for automated sample processing. As a standard technique, procedures need to be transferred between laboratories with a high degree of accuracy and precision. Its highly reproducible heating process through an automated microwave sample preparation system makes it possible to transfer accuracy and precision between laboratories.

An automated microwave sample preparation system was built using three software modules that can be used independently or in tandem. The first module is an expert system designed to aid the analyst in establishing new or selecting previously developed dissolution procedures. The second module which provides control of the measurement quality is a sample log-in system which guides the analyst through data entry, weighing, and barcoding of all samples. The third module controls the operation of a robot which attends to the material transfer and the microwave unit. The microwave control program assures reproducible reaction conditions through instrument calibration combined with feedback control of temperature and pressure inside the dissolution vessel.

---

### BIOLOGY/BIOTECHNOLOGY III

### Development of an automated reading station for microscopic slides

*J. P. Cesari and A. Cordler (Rhone-Poulenc Sante, Vitry sur Seine, France)*

Image analysis has become a commonly used tool in many research laboratories, notably in biology. This tool makes the automation of such tasks as the fastidious reading of biological tests possible, and provides, in addition, quantitification of the objects observed.

However, automation by image analysis is not sufficient for reading microscopic slides. The task of reading still requires the slides to be positioned on the microscope table; and no machine presently available on the market can do this.

The authors' aim, therefore, was to devise a completely automated system for the reading of biological tests performed with a microscope. A robot called BALfAR has been developed, which is able to position slides on a motorized microscope table, after having selected them one at a time, from a basket containing 120 slides. This system is interfaced with an Allen-Bradley Expert-Vision Image Analyzer.

This robot is presently being validated and preliminary results have shown its high level of reliability for positioning slides and for error-free reading repeatability.

### Clinical laboratory robotics in the 1990s

*R. A. Felder, J. C. Boyd, J. Savory and K. Margrey (The University of Virginia Health Sciences Center, Charlottesville, Virginia 22908, USA)*

Many creative applications of robotics in the clinical laboratory were unveiled in 1989 that forecast new future directions for this field. For example, novel robotics applications have been emerging in the DNA, critical care, drug analysis and microbiological laboratories. The incentives for the increased use of robotics in the clinical laboratory have centred around the continuing need to reduce health-care expenditures and the containment of human retroviruses such as hepatitis and AIDS.

In the diagnostic DNA laboratory a California Institute of Technology group has equipped a liquid handling robot to do PCR (polymerase chain reaction)-based diagnostic tests for sickle cell anemia and cystic fibrosis. This has allowed the normally labour-intensive DNA tests to compete with the more automated clinical analyses. Sasaki at the Kochi Medical School in Japan has created another in his series of robotic workstations which is dedicated to endocrine testing. His totally automated laboratory serves as a prototype of the clinical laboratory of the future.

Other labour-intensive areas of clinical laboratories have also been addressed by robotic engineers. For example, Medical Robotics in Lexington, Kentucky has developed a specimen processor which will centrifuge blood specimens, identify the serum/red cell interface using a laser scanner, and prepare two vials containing serum for analysis. Republic Storage Systems is offering a refrigerated robotic storage and retrieval device for medical specimens. Mobile robots created by TRC (Labmate) are being used for specimen transportation between clinical laboratory workstations. A vision-equipped version (the Helpmate) is being tested in the Danbury Connecticut Hospital for patient food-tray delivery, riding between floors on a dedicated elevator.

Although progress in robotic automation promises some relief for the burgeoning costs of a health care, attention must be focused on reliability and quality assurance. The possibility of robotic errors with grave medical and legal consequences must be weighed against the improved efficiency and reduced costs afforded by robots.

### Development of a small gantry robotic workcell for DNA filter array construction

*Tony J. Beugelsdijk and Robert M. Hollen (Los Alamos National Laboratory, Los Alamos, New Mexico 87545, USA)*

At Los Alamos National Laboratory, a primary cosmid library of human chromosome 16 has been constructed. this library consists of an 11-fold representation of the chromosome and is arrayed in microtiter plate format. A need has arisen in the large-scale physical mapping of this chromosome, to array spots of DNA from each of these colonies onto filter media for hybridization studies. We are currently developing a small gantry robot based workcell to array small spots of DNA in an interleaved format. This allows for the construction of a high spot density format filter array. This paper discussed the features incorporated into this workcell for the handling of thousands of colonies and their automatic tracking and positioning onto the filter.

---

### POSTERS

### Improving robotic assay recovery by the implementation of an alternate powder pouring procedure

*Roy C. Messaros and Stephen Scypinski (Berlex Laboratories Inc., Cedar Knolls, New Jersey 07927, USA)*

The potency analysis of in-process blends and granulations during the manufacture of pharmaceutical dosage forms is important, as the assay values obtained are often used to set the tablet or capsule fill weight of the resulting product. As the number of samples in the authors' laboratory is sometimes excessive, the manual procedure was adapted for such an analysis to the Zymark PyTechnology robotic system. During this adaptation, high variability and poor recovery of the drug substance from the blend was observed. Precision, measured in terms of the coefficient of variation, was unacceptable.

Careful examination of the robotic procedure revealed the source of the variability. During the transfer of material into the weighing tube on the analytical balance, a quantity was lost by deposition onto the top surface of the balance. This deposit was composed mostly of large particles (>425 μm diameter). In addition small particles (<45 μm diameter) were consistently left behind in the source tube after transfer. It was therefore hypothesized that if the drug was not uniformly distributed among the various particle sizes of blend material segregation during the pouring step would be occurring. This would account for the high variability and poor recovery observed.

Separation of the in-process blend material into sized fractions by sonic sifting, followed by the analysis of individual fractions for drug potency proved this hypothesis to be true. With these results the powder pouring procedure alleviated the variability and recovery problems previously experienced.

This poster described the alternate powder pouring procedure for the Zymark PyTechnology robot and showed comparative data obtained from its use against the standard pouring procedure.

### Automated solid dispensing techniques

*Michael F. Fischer, David P. McCampbell, Wayne A. Schmidt and S. W. Graves (Midwest Research Institute, Kansas City, Missouri 64110, USA)*

Since 1984, the Midwest Research Institute has been developing custom automation systems for the laboratory. One key step in many applications is the ability to accurately dispense solid materials. This poster briefly described the solid dispensing techniques used by MRI in past systems and discussed some new techniques currently in development. These new techniques include micro-metered volumetric dispensing and an air-driven cyclone-vacuum system.

### Membrane filtration for automated laboratory procedures

*Lori Southward (Gelman Sciences, Ann Arbor, Michigan 48106, USA)*

Gelman Sciences offer filters specifically designed to fit the Zymate System in a variety of membranes and pore sizes. Nylon membrane, in both 0·2 µm and 0·45 µm pore size is a very versatile, hydrophilic membrane which offers broad chemical compatibility and can be used in a wide range of applications. HPLC sample preparation and dissolution testing are only two examples.

PTFE membrane, in 0·2 µm, 0·45 µm and 1·0 µm pore sizes, offers superior chemical compatibility. The 1·0 µm pore size can be used as a prefilter for many chemicals and offers excellent flowrates. This is a naturally hydrophobic membrane and performs very well with the most aggressive solvents.

PVDF membrane, in 0·2 µm and 0·45 µm, combines wetting with aqueous solutions with broad chemical compatibility. All three types are HPLC certified for low levels of UV-absorbing extractables. The filter housing itself is polypropylene with an effective filtration area of 3·2 $cm^2$. The fluid reservoir holds 10 ml of sample and the male luer on the downstream side of the filter is an ANSI luer. The overall height of the funnel measures only 2·03 inches.

There is the capability of stacking 35 units in one dispenser tube of the Zymark Membrane Filtration PySection. This PySection can hold up to three dispenser tubes for a total of 105 filters.

In addition to the funnel filters, the Zymark BenchMate Workstation has been designed to accept the Gelman Acrodisc Syringe filters, also available with Nylon, PTFE and PVDF membranes.

The filters are available through Fisher Scientific, Baxter Scientific, Curtin-Matheson Scientific and VWR Scientific as well as the Zymark Corporation.

### Run time graphing of robotic data through use of shared data files

*Thomas Kinzelman (Zymark Corporation, Hopkinton, Massachusetts 01748, USA)*

A method for displaying a graph of robotic data while the robotic run is in progress is described. The data generated during the robotic run is written to the System V disk drive as it is generated. While the robotic run is in progress, a Lotus 123 Spreadsheet on the connected computer is actively running a program written in the Lotus Macro language. The Lotus Macro program reads the data files at regular intervals, and redraws a line graph which is constantly displayed on the monitor. The poster showed programs written in both EasyLab and in the Lotus Macro language.

### Automation of volatile organic pollutant analysis

*Bill Struve (CompuChem Corporation, Research Triangle Park, North Carolina 27709, USA)*

Analysis of volatile organic pollutants in water and soil by EPA methods 601, 602, 8010 and 8020 is done by gas chromatography. CompuChem's gas chromatographs are connected to an HP1000A computer which has a sophisticated laboratory automation system (LAS) supplied by Hewlett Packard's Analytical Instruments Division. Due to large production volume and the complex nature of these EPA methods, a hybrid of LAS and CompuChem's software was chosen.

LAS is used to collect the raw data and to determine the retention times and areas of the gas chromatographic peaks. At the end of each analysis, the software automatically loads an Image database with retention times, areas, and other information such as the dilution of the sample, sample name, and injection time, date and instrument. Compound identification and quantitation by our chemists is aided by the software which interacts with the database without further use of LAS. The hybrid system used at CompuChem reduces the time required by chemists to analyse data by a factor of 30.

### A practical solution for AgCl blinding of sensor electrode in robotic analyses for high level chloride

*Lionel H. Dahmer and Scott A. Kinzy (PPG Industries, Inc., Monroeville, Pennsylvania 15146, USA)*

Absorption of silver chloride precipitate onto the surface of a silver billet electrode is not a problem during non-automated titrations of chloride with silver nitrate. Rinsing is accomplished quite effectively with water from a simple squeeze bottle after each determination. During Zymark analytical determinations of chloride in research samples (13% NaCl), electrode blinding occurred after about five samples were processed using water as the rinse. Chloride values increased in direct proportion to the degree of blinding.

Physical rinse head changes helped somewhat, but an effective, permanent solution was then discovered to the electrode blinding problem. Based upon the solubility of silver chloride in concentrated ammonium hydroxide, it was planned to add this reagent via the MLS to each titration beaker. Before doing so, however, it was hypothesized that a dilute ammonium hydroxide rinse water solution might inhibit silver chloride absorption at the electrode. Starting with 10% ammonium hydroxide rinse water and then 1% and 0·1%, as little as 1% ammonium hydroxide totally inhibited absorption. Inhibition failed between 0·1 and 1%.

Application of 1% ammonium hydroxide rinse solution is now routinely used for these research samples. Precision data obtained using this technique showed $x = 12{\cdot}66\%$ NaCl, $s = 0{\cdot}01$, $N = 20$, compared to $x = 12{\cdot}82\%$ NaCl, $s = 0{\cdot}18$, $N = 20$ when water alone was used as rinse.

These current samples contained fairly high chloride levels. However, electrode blinding can occur to some degree when performing automated analyses of other sample matrices which contain lower levels of chloride and the use of dilute ammonium hydroxide rinse water can obviate the problem. The ultimate test of this technique will be application to saturated brine samples which contain about 26% NaCl.

### A smart robotics system for the qualitative analysis of seven metal cations

*James Blankenship, Rosie Bolen, Steven Costello, Matthew Sprouse and Frank A. Settle, Jr. (Department of Chemistry, Virginia Military Institute, Lexington, Virginia 24450, USA)*

The qualitative analysis of selected metal cations is a standard experiment in many general chemistry courses. This analysis tests the student's ability to deduce the presence or absence of ions based on experimental observations.

A Zymate II robotics system was configured to perform this experiment. The control of the system was divided between the robotics programming language, EASY-LAB, and a commercial expert system shell, VP-Expert. The analyst uses VP-EXPERT to define the parameters for the desired analysis, assembles an EASYLAB program to perform the analysis and writes the program to an ASCII file. The robot controller reads the file, performs the operations required for the analysis and writes the results to another ASCII file. The expert system then reads the results from the file and decides which ions are present, absent or uncertain. This system demonstrates the concept of integrating expert systems with laboratory robotics to obtain smart, automated laboratory systems.

### Application of the Zymate II system to the measurement of free and liposome-encapsulated doxorubicin in plasma

*J. Wayne Cowens, Paula Diegelman, Michael Reino and William Greco (Grace Cancer Drug Center and Department of Biomathematics, Roswell Park Cancer Center, Buffalo, New York, USA)*

Doxorubicin is an anthracycline antibiotic that has become part of standard treatment regimens for both hematologic malignancies and solid tumors. Because doxorubicin causes both acute gastrointestinal side effects (nausea, vomiting, diarrhea and stomatitis) and a cumulative, dose-dependent irreversible cardiomyopathy that leads to congesting heart failure, a great deal of research has been carried out to identify anthracycline formulations that retain the antitumor activity of doxorubicin but have less cardiac and gastronintestinal toxicity. Liposomes are microscopic structure consisting of one (unilamellar liposomes) or more (multilamellar liposomes) concentric lipid bilayers surrounding aqueous compartments in which both hydrophobic and hydrophilic molecules can be entrapped. A number of laboratories have described formulations of doxorubicin encapsulated in liposomes of various compositions and sizes; animal studies with these formulations have demonstrated that the acute and chronic toxicities of doxorubicin can be attenuated without decreasing antitumor efficacy. Clinical studies with these formulations have confirmed the toxicological results seen in animal systems; however, the therapeutic benefit of these formulations have not been yet proved in randomized clinical trials.

When a formulation of liposome-encapsulated doxorubicin is administered intravenously to an experimental animal or to a patient entered into a clinical trial, the drug exists in the plasma in two physically different forms: associated with the walls of the liposome and free in the plasma; the relative amounts of these two forms depends on the lipid composition of the liposome. The biological effects observed are most likely related to the relative amounts of these two forms of the drug rather than to the total drug present in plasma. In fact, in a recent clinical trial with a liposome encapsulated doxorubicin manufactured by the Liposome Company, no relationship was found between the pharmacokinetic parameters derived for total doxorubicin in the plasma and the degree of myelosuppression observed. In order to carry out further studies in Phase II and III trials, a method has been developed to separate free and liposomal associated doxorubicin in plasma; the plasma is loaded onto a solid phase extraction column packed with a stationary phase consisting of a carboxylic acid bonded to silica with a C8 spacer (Worldwide Monitoring); the liposomal associated doxorubicin is eluted with phosphate buffered saline into a tube containing TRITON X-100; the free doxorubicin is then eluted with methanol after the column is treated with HCl. The doxorubicin in each fraction is measured with a reverse phase HPLC procedure that utilizes fluorescence detection.

Because two different forms of the same compound are being measured in a milieu in which they can interconvert, a matrix of standards is required for estimating unknowns; in addition, the detector response is nonlinear over the range of concentrations that are required to be measured. Therefore, the use of this assay to estimate sets of unknowns from patients entered into clinical trials is very labour intensive. For this reason, the sample preparation required for the HPLC analysis of liposome associated doxorubicin with the Zymate II system has been automated. The samples are stored in a cooled rack; after the solid phase extraction column (BOND ELUT LRC C18, Analytichem International) is conditioned with 10 ml of 0·05M $Na_2HPO_4$ in the liquid/solid extraction station, the sample is pipetted onto the column with the pipetting hand and the doxorubicin is eluted with chloroform : methanol (2 : 1); the tubes are dried in the evaporation station and then frozen until HPLC analysis. The validation of this procedure and the comparison of this procedure with the manual procedure were presented.

### An automated method for the extraction of acetaminophen from human plasma

*Jim Fenster, Alan Hamstra and Paul Wagner (Gilson Medical Electronics, Inc., Middleton, Wisconsin 53562, USA)*

Acetaminophen N-(4-hydroxyphenyl) acetamide is an analgesic, antipyretic that has been used for years in various pharmaceutical formulations. It continues to be used in new and existing formulations by itself and with other therapeutic drugs. Therefore, the extraction of acetaminophen is a routine procedure in bio-availability and bio-equivalency studies. A method has been developed to automate the extraction of acetaminophen from human plasma using an automated sample processor (ASPEC).

The acetaminophen assay requires addition of internal standard (2-acetamindophenol) to 1 ml of plasma as a sample pre-treatment step. Solid phase extraction cartridges (C18, 100 mg) are conditioned, followed by addition of a 600 μl aliquot of sample. After selective washing and drying of the cartridge, samples are eluted into collection tubes. The collected fractions undergo a batch drydown procedure; samples are then automatically reconstituted with mobile phase and inject on an HPLC system.

The ASPEC is an x-y-z sample processor that can be used to automatically prepare samples for GC or GC/MS, or can inject prepared samples to an on-line HPLC system. Microsoft Windows-based software allows the user to easily build and store the customized extraction methods from a selection of pre-stored subroutines. Sample pre-treatment procedures, such as dilution, chemical derivatization and internal standard addition, as well as post-elution sample dilution, drydown and resuspension, are all possible with the ASPEC sample processor.

The analytical range for this acetaminophen assay is 0·2 μg/ml to 50 μg/ml. The standard curve is run in triplicate over three days. Quality control samples are assayed in duplicate with each standard curve at low, medium and high levels along with a plasma blank and a control zero. Intra-day and inter-day accuracy and precision data were presented. The precision and reproducibility of this automated procedure make it a good alternative to performing routine assays from bio-fluids manually.

### The technological and chemical aspects of the automated extraction of Zofenopril Calcium in biological fluids for GC/MS analysis

*Mark E. Arnold, Celia D'Arienzo, Eugene Ivashkiv, Mohammed Jemal and Allen I. Cohen (Bristol-Myers Squibb Pharmaceutical Research Institute, New Brunswick, New Jersey 08903, USA)*

The prodrug Zofenopril Calcium and the active drug, SQ 26,333, in biological fluids are extracted and derivatized prior to analysis by EI GC/MS. The GC/MS analysis requires extracts free of matrix interferences. The labour-intensive nature of the manual extraction prompted the automation of the extraction method on a Zymark Zymate II robot. The robotic system replicates the liquid-liquid and solid phase extraction of the manual method and provides the samples as dried extracts in autosampler vials. Development of the automated method for plasma and urine samples will be presented. Focus was placed on unique aspects of the customized hardware and software which provide reliability and versatility. Extracts provided by the automated system are of the same high quality as those produced by experienced technicians using the manual method. Results of the GC/MS validation will be discussed for plasma and urine samples. The objective of increased productivity is achieved. The robotic system increases productivity by more than 100%, while using less technician time in sample preparation.

### Automated iodination of proteins and peptides using a Zymark robot

*Brian Fletcher, Anthony Chen and Beth Hutchins (Departments of Medical and Analytical Chemistry, Biological Chemistry Section, Genentech, Inc., South San Francisco, California 94080, USA), and Gordon Stelling (Environmental Health and Safety, Genentech, Inc., South San Francisco, California 94080, USA)*

An automated procedure for the iodination of proteins and peptides has been developed using a Zymate II robot with a dual purpose hand (100 μl syringe and gripper functions). The menu-driven system has been programmed to perform iodinations using the Chloramine T, iodogen, and lactoperoxidase methods. After the reaction is finished, the robot transfers the mixture to a small gel filtration column where the tracer is collected in one fraction separated from the free iodine.

The major advantage of this system is that it isolates the user from most of the gamma radiation normally encountered during a manual procedure. Careful consideration was given to the safety aspects of the system. Absorption of free radioactive iodine by robot surfaces was minimized with the use of a removable film, an inexpensive and practical solution, and monitoring programs were established. Fume hood function was found not to be compromised by the presence of the system.

Interaction with health and safety personnel, system optimization, issues related to safe hardware maintenance, and documented training of users are all key components of the effort necessary to implement this type of system.

The automated iodination procedure is simple to use and has been very positively received by users.

### Automated robotic extraction of proteins from plant tissue samples

*Thomas B. Brumback, Jr. (Pioneer Hi-Bred International, Plant Breeding Division, Johnston, Iowa 50131, USA)*

The use of recombinant DNA techniques to introduce foreign genes into crop plants has increased the need for techniques to assess the presence of such genes. Typically, protein products from reporter genes are assayed to detect the presence of foreign genes. The most laborious step of such assays is the extraction of the proteins from raw plant samples.

A custom robotic system (Bohdan Automation, Chicago) was developed to automate the extraction of proteins from plant samples. Leaf or callus material (5 to 25 mg) is presented to the robot in 1·5 ml Eppendorf tubes, the system performs buffer dispensing, grinding, centrifugation, and pipetting unit operations, and a cleared supernatant is delivered in a 96-well microplate format for subsequent analysis.

The system consists of two overhead x-y-z arms, an Allen Bradley (Cleveland) programmable logic controller, a microcomputer for user interface and control, and several custom peripheral devices for sample handling and grinding. The system is housed on a 4 × 5 ft table and contains a back-up power supply and a refrigeration unit to prevent sample degradation. The unit operates in a batch mode and is capable of processing more than 100 samples per hour. The design, development and performance of the system were discussed.

### An automated instrument for the performance of enzymatic DNA sequencing reactions

*Jonathan D'Cunha, Bennett J. Berson, David A. Mead, Robert L. Brumley, Jr., Paul R. Wagner and Lloyd M. Smith (Department of Chemistry, University of Wisconsin, Madison, Wisconsin 53012, USA)*

The Human Genome Initiative is a massive international project to determine the detailed nucleotide sequence of the human and other genomes. One critical focus of current efforts in this area is the development of new automated technologies to reduce the cost and expense of sequence analysis. The construction and use of an instrument which automates one aspect of sequence analysis, the enzymatic extension reactions, was described. The instrument is based upon a Gilson Model 222 HPLC Autosampler to which two temperature controlled assemblies have been added. One assembly holds the reagents (maintained at 0–2 °C) and the second assembly holds up to five 96-well microtiter plates, in which the sequencing reactions are performed. The autosampler adds the reagents to each of templates and the temperature is raised from 0–2 °C to a temperature appropriate for the enzymatic extension reactions. Up to 96 DNA templates can be processed for either radioactive or fluorescence-based sequence analysis in a 3 h period. The accuracy of the resultant sequence data is equivalent to that obtained manually. The system is simple, flexible and readily adapted to the use of new polymerases or modified experimental protocols.

### Robot accuracy for small DNA sequencing reactions

*John Shigeura (Applied Biosystems, Foster, California 94404, USA)*

To be economically feasible, the cost of large-scale DNA sequencing must drop from the currently estimated $3–5 per base pair of finished sequence to approximately $0·50 per base pair. Among the many improvements needed to achieve such a cost reduction is the scaling down of the volume of DNA sequencing reactions. Cutting down the reaction volume decreases the size and expense of the instrumentation, as well as the volume and cost of the reagents consumed, and may simplify some chemistry protocols.

A robot dedicated to performing small DNA sequencing reactions (volumes of 10 μl or less) must possess an accuracy of motion sufficient for fine manipulations. The computation indicates that, to manipulate reaction volumes of 1–2 μl, a robot should achieve a circular positional accuracy in the range of 0·2–0·6 mm diameter. This poster described a test for robot accuracy and presented test data from a robot currently being developed to perform small-volume DNA sequencing reactions.

### Applications of the BenchMate in automated sample preparation for HPLC

*W. Jeffrey Hurst (Hershey Foods, Hershey, Pennsylvania 17033, USA), and Brian G. Lightbody (Zymark Corporation, Hopkinton, Massachusetts 01748, USA)*

The BenchMate is a member of the instrument category that can be classified as robotic workstations. One of the primary uses of robotics has been sample preparation for chromatography. This presentation presented a number of examples on the use of this instrumentation in preparing samples for HPLC analysis. The applications that were presented ranged from unique methods for sugar analysis using Freon TF and water, to applications suitable for general laboratory usage such as automated derivative formation in amino-acid analysis.

### Automated chemistry workstation for tablet extraction and analysis

*A. Martin (Source for Automation, Inc., Milford, Massachusetts 01757, USA)*

Source for Automation's Automated Chemistry Workstation for Tablet Extraction and Analysis incorporates unique extraction technology, along with PG-based workstation control of methods and test data. Designed to perform dose uniformity testing in real time with on line reporting of results, this workstation is compatible with most HPLCs or UV/VIS equipment.

In pharmaceutical analysis, the assay of the active ingredient(s) of a tablet starts with an extraction of the compound(s) of interest from the tablet. To achieve this, the tablet must disintegrate for the extraction fluid to reach the inner parts of the tablet.

The unique design of the extractor causes the breakdown of the tablet, releasing the active ingredients into solution in minutes. The sample is then diluted, with volumes gravimetrically verified. An aliquot of the sample is passed through a 25 mm 0·45 micron filter into a fixed loop injector, which introduces it directly into an HPLC or UV/VIS.

Designed for high throughput, this workstation cuts sample preparation, data collection, and reporting time. Data is transferred electronically, generating reports in accordance with United States Pharmacopeia (USP) guidelines.

### Robotic basket dissolution with concurrent off-line analysis

*David G. Walter and Olu Aloba (Parke-Davis Pharmaceutical Research, Warner Lambert Company, Morris Plains, New Jersey 07950, USA)*

A Zymark Basket Dissolution System was re-plumbed and re-programmed using 'Concurrent EasyLab', to enable off-line and on-line sampling. Consecutive single-point and multi-point sampling of immediate release tablets were carried out, with samples stored off-line and subsequently injected onto the HPLC column whilst the robot washed and filled the vessels for the next dissolution run. The system compares favourably with the manual procedures and meets USP specifications. As a result of this effort, the system is now capable of dissolution testing with paddles or baskets, UV or HPLC analysis and on-line or off-line sampling.

### Robotic dissolution testing of microencapsulated dosage form analyses in the pharmaceutical industry

*John A. Steichen (Adria Laboratories, Quality Control Lab., Columbus, Ohio, USA)*

Recent developments in robotics have been applied to fully automate tablet/capsule dissolution testing. Dissolution testing is one of the most common, labour-intensive procedures, in the pharmaceutical laboratory. The Food and Drug Administration, as per USP requirements, requires dissolution profiles to be determined on numerous oral dosage forms.

Fully automated dissolution testing generally requires the use of robotics to completely integrate all the sample preparation steps with analysis and reporting. The system described in the poster automatically fills the vessels, test media temperatures, places samples in the vessels, analyses the samples, washes the vessels, reports sampling times, repeats the process for the required number of samples and shuts itself down. All the steps rigidily conform to the United States Pharmacopeia (USP) guidelines.

Furthermore, this system fully automates the UUSP basket method of dissolution testing. Performance data were presented to demonstrate precision and accuracy for such critical factors as media volume delivery, temperature control, vessel and sampling carry-over and sampling dilutions.

Data profiles were presented for a specialized extended release microencapsulated potassium chloride product, showing percent dissolved versus specified time periods. A novel way for testing the tiny encapsulated potassium chloride beads in the USP baskets was also presented (patent applied for). Comparisons of manual versus robotic procedures were provided to show quality of results and validation.

### Semi-automated analysis of sustained release capsules using the Zymate II robot

*Kaarlo Hentila (Lederle Labs, American Cyanamid Company, Pearl River, New York, USA)*

The Zymate II paddles EasyLab dissolution software was modified to produce a semi-automated basket procedure for the analysis of a 24-hour sustained release capsule product using dissolution tanks from Van-Kel. The developed procedure requires minimal human intervention at the sample introduction point. Afterwards, robotic analysis of these samples is performed using a Beckman DU-30 UV/VIS spectrophotometer at 1, 4, 8, 11 and 24 hour sampling time points. Sample readings were compared to standard readings taken just prior to each sampling time point. A printed report of all calculated results is generated which takes into account media volume loss over time due to evaporation.

According to the procedure, the operator manually lowers the basket-shaft assembly containing the capsule into the media to a pre-determined mark when prompted by the robot. Next, the shaft chuckhead is tightened, and the clutch is engaged to begin the basket rotation. This process is repeated until all capsules to be analysed have been dropped. When analysis of the samples is complete, the operator manually raises the basket-shaft assembly back to the original position, and starts the robotic wash program which begins a spray wash of the dissolution vessels.

Since five blanks and five standard absorbance measurements are taken for this application, ten storage beakers containing 80 ml of blank or standard solutions were made available to the robot for these readings. Blank and standard positions were incremented through the software after every time point.

The results produced by this robotic procedure compared almost exactly with results generated manually. The system is intended to be a good cost saver and we are pleased to present it here.

### Laboratory robot for the microbiological assay of neomycin

*Donna S. Miller and Sandra L. Grosso (Bristol-Myers Squibb Company, New Brunswick, New Jersey 08903, USA)*

A Zymark PyTechnology I laboratory robot was developed to perform the microbiological assay of neomycin by the turbidimetric method. Custom devices designed for this application include a circulating constant temperature water bath, a custom liquid dispensing hand plumbed to a rapid dispenser, and a self-sanitizing cannula hand for inoculating the test medium. Following initial sample extractions and bench setup, the robot completes the assay unattended. The robot dilutes standard and samples, prepares and dispenses the inoculated medium, incubates the assay tubes, quenches bacterial growth and then measures the absorbance of each sample using a spectrophotometer. Precision is improved because all samples are treated identically and with strict control of each assay step. The automated method provides for greater laboratory productivity because the system operates unattended and during off-shift hours.

### Microbial enumeration of broths and soils

*Walter C. Gates, Jr. and Donald G. Martin (Texaco, Inc., Beacon, New York 12508, USA)*

A Zymark system, in use in an open laboratory for other purposes, has been expanded to prepare serial dilutions and to transfer aliquots to Petri dishes for enumeration. The incubated dishes appear very good. For reactor broths, relative errors in dilution and in plating are each reduced 50%. For solid slurries, the robotic procedure has at least marginally less error than the manual one. No contamination is introduced by the procedures, but the time needed is longer. To maintain the ability to do the original work, system capacity is limited to 40 plates per intervention.

### Robotic vision system for measuring zones of inhibition in a microbiological standard curve cylinder plate assay

*W. D. Vanden Bosch, K. M. Jenkins, C. S. Foran and K. Tsuji (The Upjohn Company, Kalamazoo, Michigan 49001, USA)*

The measurement of zones of inhibition in microbiological plate assays has been a tedious manual procedure. Each plate was presented to a measuring device by an analyst who then measured each zone one at a time, recording the measurement on a record or entering it into a computer system. The introduction of vision systems enabled the zones on a plate to be measured at one time, but still required the presence of an analyst to present the plates to the system, read the zones, store the data for report generation, and discard the plates at the conclusion of the analysis[1].

A flow chart was prepared of the activities required for the zone measurement process. A vendor was contracted to build a robotic system that would carry out the plate assays zone measurement procedure. The system consists of three robots organized on a platform with a plate stacking device, a label sensing device, and a barcode reader.

Barcodes are prepared and affixed to the plates prior to placing cylinders on the plates. The plates are spotted and incubated overnight. The plates are placed in the plate stacker for presentation to the measurement system. The system is initialized using an IBM AT computer. The robotic system removes the cylinders, identifies the plate and sample numbers and orients the plates for presentation to the vision system. When readings of the zones are completed, the system discards the plate into a disposal container.

The robotic vision system enables cylinder plate assay zones of inhibition to be read accurately and quickly without the presence of an analyst.

#### Reference

1. Foran, C. S., Evans, D. M. and Tsuji, K., *Automated Machine Vision-Image System for Measurement of Antibiotic Zone of Inhibition* (in preparation).

### A robotic system for preparing and analysing paediatric suspensions

*L. J. Lorenz, R. J. Lykins and J. Zynger (Eli Lilly and Company, Indianapolis, Indiana 46285, USA)*

Reconstitutable paediatric antibiotic suspensions are complex samples to handle. These materials exist as a dry powder to which water is added to form a suspension at the time that the drug is dispensed. These types of materials tend to be very thick and sampling is a problem even under ideal conditions. To complicate the issue further, the kinetics of drug degradation are greatly enhanced upon exposure of the drug to the water in the suspension. Thus, the analysis of the drug at the time of preparation is critical.

A robotic system has been developed to reconstitute paediatric suspensions and to subsequently process samples for the appropriate assays. This system withdraws multiple samples from the prepared suspension to determine the dose potency of the drug by HPLC techniques. Additional samples are withdrawn and diluted in an appropriate manner to determine degradation products by HPLC gradient techniques, ion chromatographic procedures, and size exclusion procedures. Samples are also taken to determine preservative content by HPLC techniques. The system also determines the density, viscosity, and pH of the paediatric suspension.

The robotic system used in this application consists of two robotic arms which undergo co-ordinated activity controlled by a master computer serving as the system controller. The system also communicates to a laboratory data system for the collection and processing of the chromatographic data. The controlling computer verifies and documents all critical activities of the robotic system.

### Automation of sequential impurity and potency assays of sterile drug products

*Lisa D. Martin (ICI Americas, Inc., Wilmington, Delaware 19897, USA)*

Impurity and potency assays are part of a series of stability-indicating tests conducted at regular intervals on new drugs. A PyTechnology robot has recently been installed to perform both assays on a sterile drug product. A flexible system was designed to accommodate various drug dosages, packaged in four different sized, septum-sealed injection and infusion vials. The sample, contained in a septum-sealed vial, is constituted at a vial piercing station. After dissolution, an aliquot of the dissolved sample is transferred to a test tube for further dilution. Degradation products and impurities are identified and quantitated using reverse-phase HPLC. Further dilution is required prior to sample injection for drug potency and identity determination by HPLC. Two detectors in series provide optimum conditions for both assays with a single HPLC unit. In this poster, the robotic system used to prepare and introduce sterile samples to the HPLC for impurity and potency assays were described.

### Fully automated HPLC system with function of self-diagnosis

*J. Matsushita, K. Kawata, Y. Okamoto and T. Yasuda (Fujusawa Pharmaceutical Co., Ltd, Osaka, Japan)*

A variety of different drug substances and drug products are being tested daily by HPLC in the authors' laboratory. Although an HPLC method is widely applied to the quality control of pharmaceutical products, the HPLC procedure comprises some labour-intensive operations: sample preparation, instrumental conditioning, injection and measurement, data handling, and report generation. The instrumental conditioning, in particular, requires a lot of operator's judgement and confirmation, so this step has been recognized as the most difficult part for analysing multiple samples successfully and automatically.

The aim was to construct a fully automated HPLC system – an intelligent HPLC with the function of self-diagnosis, which can adjust instrumental conditions automatically. The HPLC was coupled to a Zymark laboratory robot and a data processor through a distributed computer control network. Additionally, software was developed for this system.

The automated HPLC system can be started by indicating a code name of test specimen to the pre-programmed method file in the host computer, then instrumental conditioning and sample preparation can be performed simultaneously. The function of self-diagnosis enables the HPLC to check the stability of base line (noise level and drift), and to adjust the flow rate and detector sensitivity automatically. The sample preparation can be achieved by the Zymate robot with three interchangeable hands, a sonicator, a stirrer, solvent dispensers, a filtration device, a pipetting device, and a vial filling device. Furthermore, the robot is capable of injection of the prepared sample solutions by setting up the vial to the autosampler during the sample preparation process according to the direction of the host computer. The system can also generate a suitable data sheet through the automatic data processor.

Utilization of this fully automated HPLC system is expected to increase the productivity of the authors' laboratory and free analysts from routine tasks.

### Robotic system for general liquid dosage form cough syrup with on-line HPLC analysis and report generation

*V. W. K. Lam and J. K. S. Lee (Parke-Davis Division of Warner Lambert Canada, Inc., Scarborough, Ontario, Canada M1L 2N3)*

A Zymark PyTechnology robotic system with System V controller was interfaced with a high performance liquid chromatograph (HPLC) and Hewlett Packard 3393A integrator. The whole process, from sample preparation to report generation, involves no manual operation after initial setup and output.

The robot transfers the samples, adds solvent for extraction and then oversees the vortexing, sonication of the samples. The samples are filtered using depth filter and injection into the HPLC. The HP3393A integrator collects the data and transmits the results to the robot which prints out the final report.

The major advantages are: (1) the robot runs totally unattended with very little start-up time; (2) the smart interface between the robot and the HP3393A integrator produces sample ID details and final results; (3) the system can analyse many different products automatically using programmable UV detector and HPLC pump, solvent selector switching and column switching technology; (4) the comparisons of manual and robotic results show no difference.

### Multiple human cytokine immunoenzymetric assays performed in parallel on the Biomek 1000

*Donald K. McRorie (Beckman Instruments, Inc., Palo Alto, California 94304, USA), and John S. Abrams (Department of Immunology, DNAX Research Institute, Palo Alto, California 94304, USA)*

The ability to quantify cytokines, a new and expanding family of immunomodulatory factors, in patient samples is an important tool in uncovering the underlying mechanisms in many diseases. Immunoenzymetric assays provide a useful alternative to bioassays which often lack the specificity of monoclonal antibody-based immunoassays. These assays are performed by sandwiching the cytokine molecule between a capture antibody coated on a solid-phase and an enzyme-labelled detecting antibody to quantify the presence of these factors in patient specimens. In order to investigate cytokine profile patterns, one sample is tested in several different cytokine immunoassays; microtiter plate-based assays facilitate sample handling and analyses. These procedures have been automated using the Biomek 1000. Initially, the same sample was screened in a panel of different cytokine immunoassays (30 samples per plate). Those samples which were positive were then assayed at three different dilutions (10 samples per plate) to confirm specificity and quantity based on parallel line assay design, using recombinant standards. Addition of the side loader allowed walkaway capability for this series of assays permitting the simultaneous screening of the same sample in as many as seven different cytokine assays.

### The S-2251 chromogenic assay for the measurement of enzymatic activity of recombinant novel plasminogen activator: development and application on the Zymate microplate management system

*Joe Franchetti, Dwight D. Moore, Shing Mai and Benjamin J. Del Tito, Jr. (Smith-Kline Beecham, King of Prussia, Pennsylvania 19406, USA)*

The Zymate Microplate Management System has been adapted to completely automate the S-2251 assay for the measurement of enzymatic activity of recombinant novel plasminogen activator. In this assay, recombinant novel plasminogen activator converts plasminogen to its active form, plasmin, in the presence of fibrinogen. Plasmin then cleaves the chromogenic substrate (S-2251, or D-VAL-LEU-LYS-p-nitroanilide) to produce a p-nitroaniline which is measured at a wavelength of 410 nm with a reference of 490 nm. This automated procedure eliminates errors caused by manual reagent additions and sample dilutions, while decreasing total analyst time.

The manual procedure, which was developed in Smith-Kline Beecham's Analytical Development Laboratories, was modified and validated such that the System V Microplate robot could easily accommodate this assay. The procedure developed on the System V consists of the following steps: (1) addition to diluent to the sample dilution plate(s) and to the standard plate(s); (2) two-fold serial dilutions of the samples; (3) preparation of a standard curve; (4) addition of substrate to the assay cocktail; (5) addition of cocktail to the assay plate(s); (6) incubation of the plate(s); (7) cessation of enzymatic reaction by addition of acetic acid; and (8) spectrophotometric determination via a microplate reader. The accumulated raw data is sent to a VAX mainframe and collected utilizing proprietary software and RS/E. Regression analysis was developed in-house to evaluate the data. The total time required for the Zymate microplate system complete the assay is 1 hour to one plate of eight samples.

Once the application was completed, system validation was performed. This consisted of validating the three Reagent Addition Modules, the eight channel pipette hand, the one milliliter pipette hand, and the incubators. This was followed by assay validation and comparison with the manual procedure. These results were discussed in detail during this poster presentation.

### An automated ELISA system for cross-reaction testing of monoclonal antibodies to commercial seafood products

*Stephen M. Mayfield, Ronald C. Lundstrom and Margaret M. Russell (National Marine Fisheries Service, Gloucester Laboratory, Gloucester, Massachusetts 01930, USA)*

The National Marine Fisheries Services' laboratory produces monoclonal antibodies for use in species identification of seafood products and for some time had been grappling with the problem of cross-reaction testing. For monoclonal antibodies to be useful, their cross-reactivity must be characterized against hundreds of commercially important sea food species. The answer was laboratory automation in the form of a Zymark† microplate system setup to perform ELISA procedures.

During each experimental run the laboratory robotics system is programmed to assay 24 monoclonal antibodies against 96 antigens. The antigen solutions are accessed from 1 ml tubes stored in 8 by 12 microtiter format racks using a 12-channel pipetting hand. The monoclonal antibody solutions are pipetted from divided reservoirs with an eight-channel pipetting hand. Other solutions required for the ELISA procedure are added to the assay plates through eight-channel manifolds at a reagent addition station. Between each step in the ELISA procedure the assay plates are washed to remove unbound reagent solution. Plates are read photometrically and the results are transmitted via a serial interface to a spread sheet.

The microplate system has allowed the authors' to increase throughput approximately three-fold over ELISA procedure performed manually. Automation has also resulted in a dramatic improvement in the confidence levels of data.

† Mention of trade names or commercial firms does not imply endorsement by the National Marine Fisheries Service, NOAA.

### Automatic lab leading experience

*E. Muttoni (ENICHEM ANIC, Porto Torres, Italy)*

The author presented an integrated unit which controlled two operating robots (working simultaneously). The unit can effectively complete handling of the sample (identified through a barcode), storage of results and issue of reports.

System features include:

(1) Ph and conductivity detection.
(2) Acid based potentiometric analysis on aqueous or organic matrices.
(3) Moisture determination according to the Karl-Fischer method.
(4) Colorimetric analysis on water samples.
(5) Sample preparation of secondary samples for chromatographic analysis.

It is capable of handling 250 samples a day, providing around 500 results.

The system is composed of a minicomputer for system management, a section for management of sample transfers, and two benches on which the two robots work for carrying out analysis.

### Feedback control of heating and cooling in custom PyTechnology reactor workstations

*Susan D. Boettger (Bristol-Myers Squibb Company, Chemical Process Research Department, Syracuse, New York 13221, USA)*

The Chemical Process Research Department of Bristol-Myers Squibb currently uses a custom Zymark PyTechnology system to optimize organic reactions. Reactions are run in one of four Pierce Reacti-Therm heating/stirring modules attached to PyPlates. The first goal was automatic control of the heating function of each module. Each heater is equipped with low and high temperature dials and a switch designating a low (25–75 °C) or high (75–150 °C) temperature range. The low resolution on the dials makes precise temperature control very difficult. In addition, manual intervention would be required to change temperatures during a run. This poster illustrated the control of workstation heaters via feedback from custom platinum RTD probes and voltage regulation by the Zymark Power and Event Controller (PEC).

The second goal was development of an automatic workstation cooling system. Ten channels were drilled into each workstation block for circulation of cryogenic fluid. Vial temperature was coarsely controlled by the bath temperature and flow rate. Fine-tuning of the low temperature system was achieved by PEC control of a flow-directing valve based on RTD probe feedback from the reactor block.

